# Experimental analysis of PTFE and PTFE/PEEK composites for ball bearing applications

**DOI:** 10.1038/s41598-025-31212-w

**Published:** 2025-12-07

**Authors:** Dhruv Deshwal, Sachin U. Belgamwar, Siddappa I. Bekinal

**Affiliations:** 1 Department of Mechanical Engineering, Birla Institute of Technology and Science Pilani, Pilani Campus, Pilani, Rajasthan 333031 India; 2 Department of Mechanical and Industrial Engineering, Manipal Institute of Technology, Manipal Academy of Higher Education, Manipal, Karnataka 576104 India

**Keywords:** PTFE, PEEK, Reinforcement, Bearings, Wear rate, Vibration, RCF, Aerospace engineering, Mechanical engineering

## Abstract

Polytetrafluoroethylene (PTFE) composites are extensively utilized in tribological applications. In bearing applications, they have found use in journal bearings, bearing pads, lubricants, and other related contexts. However, the applicability of PTFE in ball-bearing systems has yet to be thoroughly investigated. This article begins by examining the wear behaviour of both pure PTFE and 15% PEEK-filled PTFE through ball-on-disc tests using silicon nitride balls. Subsequently, ball bearings incorporating silicon nitride balls were fabricated and subjected to free run and rolling contact fatigue tests. The findings indicate that pure PTFE is unsuitable for ball-bearing applications, whereas PEEK-filled PTFE may be viable for low-load and low-RPM applications.

## Introduction

PTFE has emerged as an excellent material used in engineering applications^1^. The material is a semi-crystalline polymer consisting of a series of carbon atoms, with each atom connected to two fluorine atoms. The fluorine atoms positioned around the carbon chain give the molecule excellent stability and low reactivity. PTFE is not chemically reactive and has very low water absorption, leading to outstanding dimensional stability^2–5^. It has excellent tribological properties, a low coefficient of friction (COF), and excellent self-lubricating properties^6–8^. Furthermore, it has high thermal stability^9,10^ and can be used in the widest temperature range^11^. The crystalline melting point is about 327–342 °C^11,12^ and can retain its mechanical properties in the temperature range of –260 to + 260 °C^13^, which can replace the metal components for low-temperature applications^14^. Due to its lower COF, it is used as a solid lubricant^15^ in many applications, which include bearings^16,17^, lubricants^18^, precision^19,20^, refrigeration^21^, etc. The long (CF_2_-CF_2_)_n_ chains in PTFE have low shear strength, resulting in a low COF during sliding^22^. Adding filler materials can improve PTFE’s wear resistance, but the effectiveness of the composite depends on factors like the type and amount of filler used^23^.

PTFE exhibits high wear rates and low load-bearing capacities^24^; however, reinforcement of the filler material has effectively addressed these issues. The reinforcement not only improves PTFE tribological performance but also improves its mechanical and thermal properties^24^. The primary reinforcement added to the PTFE matrix includes metals^25–27^, ceramics^28–30^, polymers^31,32^, fibres^33,34^ and carbon-based materials^35–37^. The polymer reinforcement in PTFE enhances its wear resistance, mechanical strength, and durability.

Polyetheretherketone (PEEK) is among the significant polymer-based filler materials that researchers have explored to enhance the service of PTFE^38,39^. PEEK is a thermoplastic polymer introduced in 1978 by Imperial Chemical Industries^40^. It belongs to the polyaryl ether ketone (PAEK) family^41,42^, and has a strong molecular structure with functional groups that enhance intermolecular attraction^43^. In a study, PTFE reinforced with 50 wt% PEEK showed a COF of 0.111, whereas unfilled PTFE had a COF of 0.132. Furthermore, PTFE with 32 wt% PEEK had a wear rate of 2 × 10^−9^ mm^3^/Nm, making it more wear-resistant than unfilled PEEK and PTFE^44^. This shows that the PTFE/PEEK composite would be an excellent combination for tribological applications. In recent times, the use of polymeric materials in bearing applications has become increasingly popular, substituting for metal usage to some extent. The use of PTFE composites as a bearing material can offer a lightweight, lubrication-free, and corrosion-resistant bearing, which is widely used in the food industry, medical technology, and automotive sectors. However, they still face challenges such as low bearing capacity and thermal conductivity^22,45,46^. In the past, researchers have manufactured and tested PEEK-based ball bearings to calculate their load-bearing capacity^16,47–55^. Along with these, several studies have compared PEEK and PTFE composites for bearing applications^56,57^.

A surge in the use of PTFE in mechanical engineering applications has been observed over the past few decades, and its use in bearing applications has been widely accepted. However, its feasibility in the ball-bearing application is very limited. Our previous research has explored the usage of 25% glass-filled, 40% bronze-filled PTFE, and pure PTFE for ball-bearing applications. The research indicates the potential to enhance PTFE’s bearing performance by incorporating reinforced filler materials. The proposed research uses PTFE filled with 15% PEEK (PEEK15) to investigate its applicability in ball-bearing applications. Initially a tribological test was conducted though ball-on-disc test and its tribological characteristics were evaluated. Then a single-row deep groove ball bearings with silicon nitride balls (Si_3_N_4_) were fabricated. The fabricated bearing testing is done through free run and rolling contact fatigue test. During this test various vibrational characteristics were measured with the help of the DAQ system.

## Experimental details

### Material

In this study, the suitability of PTFE and 15% PEEK-filled PTFE was checked for a single-row deep groove ball bearing application. A circular rod of 40 mm diameter and 200 mm length is procured from Hindustan Nylon, Miraj, India. The balls used in the test and bearings are Si_3_N_4_ balls, which were procured from N Gandhi & Co., Mumbai, India. The material properties are specified in Table 1.

**Table 1:** Properties of materials.

Properties	Pure PTFE	PTFE + PEEK (15%)	Silicon nitride
Designation	PTFE	PEEK15	Si_3_N_4_
Density (gm/cm^3^)	2.14	1.94	3.20
Tensile strength (MPa)	21.80	8.43	700
Elongation	260%	190%	–
Hardness	54 Shore D	59 Shore D	2477HV
Service temperature (℃)	260	300	1100

### Fabrication of bearings

**Fig. 1:**
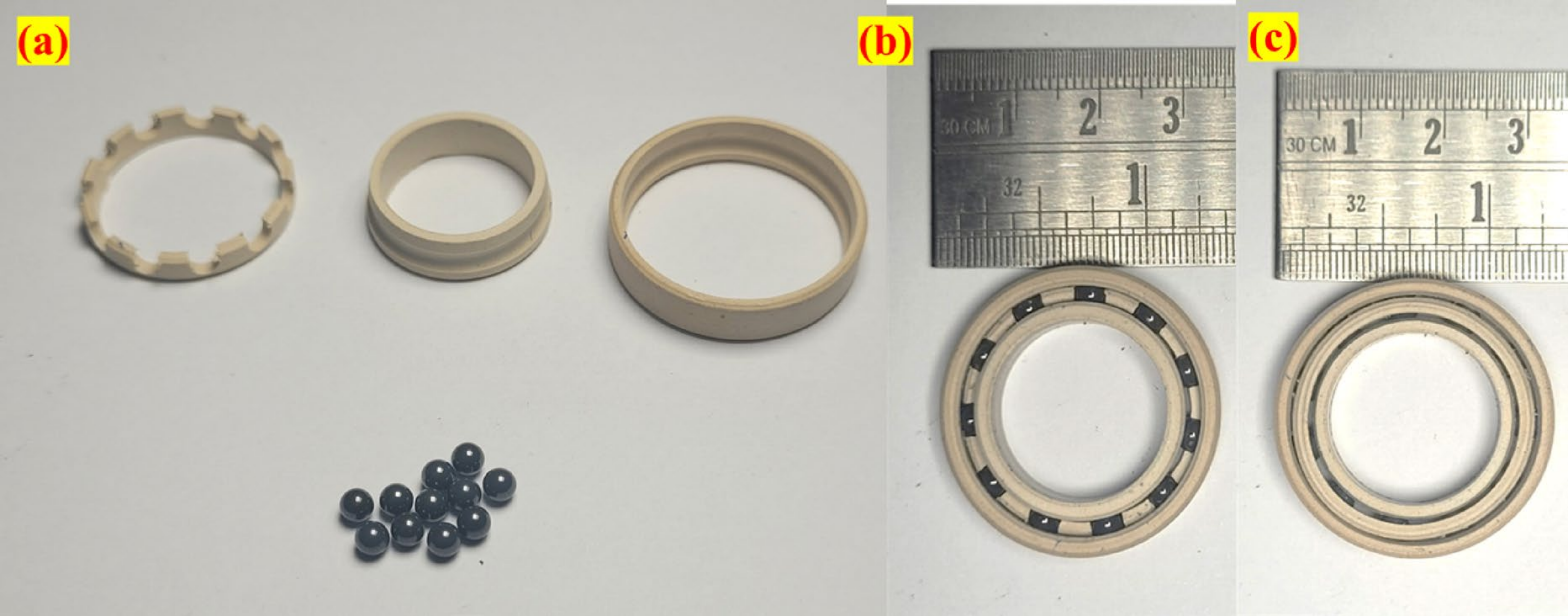
(**a**) Fabricated PEEK15 bearing parts and assembled PEEK15 bearing (**b**) front view (**c**) rear view.

The fabrication process is performed on a multi-axis CNC machine to produce the inner ring, outer ring, and cage of the bearing, all of which are made from the same material. Figure 1a showcases the PEEK15 bearing parts along with the silicon nitride ball of diameter 5/32 inch, and Fig. 1b, c shows the front and rear views of the assembled bearings. The design of the fabricated bearing is inspired by IGUS bearings, with dimensions equivalent to those of the SKF 61804 grade single-row deep groove ball bearing. The bearing has a bore diameter of 20 mm, an outer diameter of 32 mm and a width of 7 mm. The bearing conformity ratio is 0.52, and 11 balls are used in the bearing.

### Testing methods

#### Ball-on-disc test

**Fig. 2:**
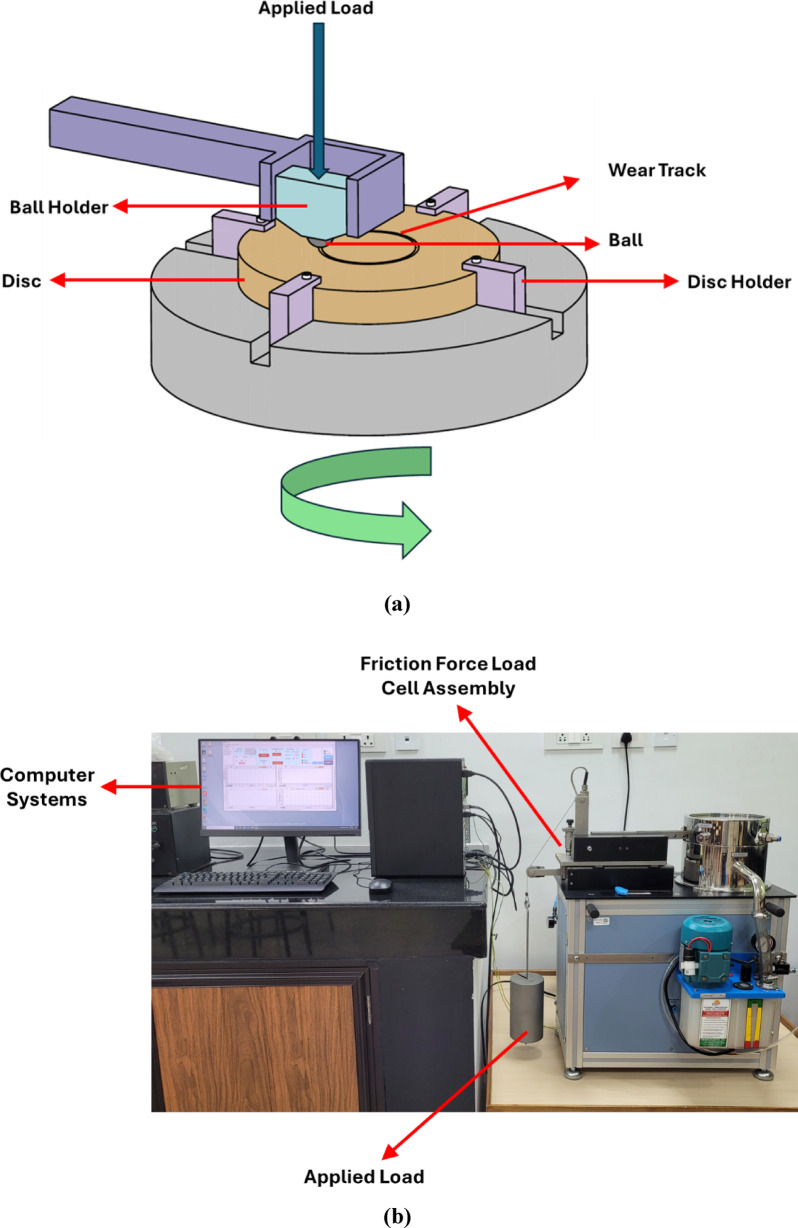
Ball-on-disc test setup (**a**) Schematic representation (**b**) Actual setup.

The tribological characterization was carried out on the ball-on-disc setup in the Ducom TR20 NEO series friction and wear monitor, as shown in Fig. 2. The test is conducted to calculate the COF and wear rate of PTFE and PEEK15 with a Si_3_N_4_ ball of 6 mm diameter. For the test, discs 40 mm in diameter and 8 mm thick were fabricated from PTFE and PEEK15 materials, and their properties are listed in Table 1. The disc’s surface was polished with 1500-grade sandpaper to remove the machine marks. Later, the roughness of these discs was measured using the Mitutoyo profilometer, yielding values of 0.77 µm and 1.22 µm for the PTFE and PEEK15 discs, respectively. The disc was rotated at 800 RPM with a 20 mm wear track. The test was conducted in dry conditions at 25 °C and 35% relative humidity. Friction and specific wear rate values were obtained for loads ranging from 25 to 75 N, with step loading of 25 N. The field emission scanning electron microscope (FESEM) was used to analyse the wear track formed on the disc. Equation (1) was used to calculate the specific wear rate.

1 $$WR = \frac{\Delta V}{{F.D}}$$

where, ΔV is volume loss (gm/mm^3^), F is normal load (N), and D is the sliding distance (m).

#### Free run test

**Fig. 3:**
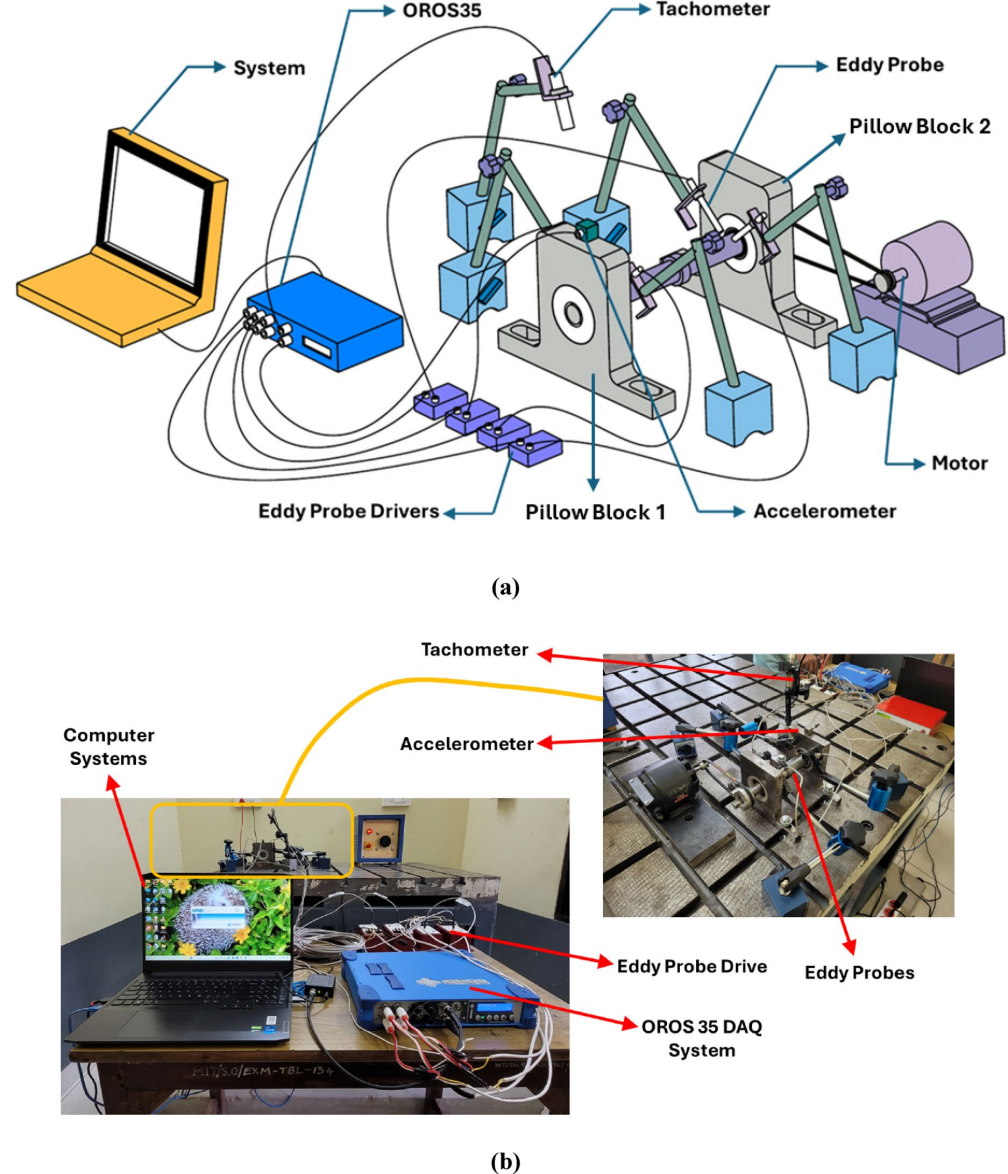
Free run test (**a**) schematic setup^58^ (**b**) actual setup.

The rotor was supported by the fabricated bearings, and its dynamic characteristics were measured through a free-run test setup, as shown in Fig. 3. In the test, a set of bearings is used to support a 170 mm long shaft. Then, the bearings were supported in the pillow blocks, which restricted the shaft’s axial and radial movements. The motor (147 W and 50 Hz) was coupled to one end of the shaft using a belt-drive mechanism. The DAQ (OR35-INST) system is a 6-channel system that utilises the ORBI gate and NV-gate software for recording signals. The post-analysis of the recorded signal is performed with the aid of a computer system. The sensors used during the test consist of an accelerometer (1-axis ORAC-DCC-D23) for measuring vibration amplitudes, which was placed over the pillow block, and an optical tachometer (ORAC-TACMM-001) for the speed of the rotor. Furthermore, the vibration characteristics are measured using eddy probe sensors (MTN/EP080). These eddy probes were used in 2 sets, with each set placed near both pillow blocks. The eddy probes, accelerometer, tachometer and PC are connected to the DAQ system.

#### Rolling contact fatigue test

**Fig. 4:**
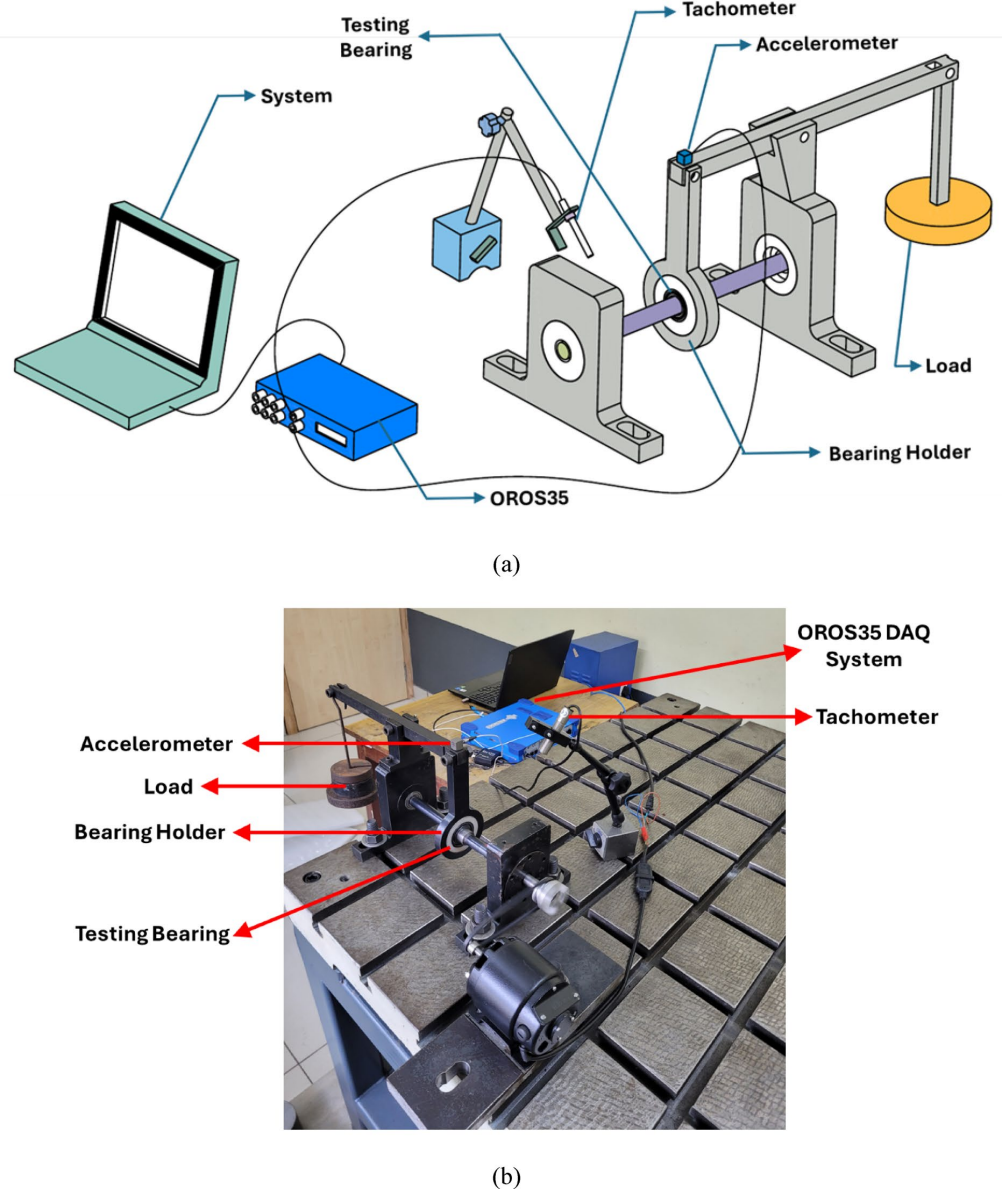
Rolling contact fatigue test (**a**) schematic setup (**b**) actual setup.

The RCF test was conducted on the fabricated bearings to determine their bearing capacity. The bearing is tested up to 1 million cycles and subjected to three levels of loading at a constant RPM of 800. The bearing was subjected to loads of 25, 50 and 75 N. According to the loading system with a lever, the actual loading conditions on the test bearing were 36, 72, and 108 N, respectively. Initially, the bearing was subjected to 36 N for the first 0.25 million cycles, then to 72 N for the next 0.5 million, and finally to 108 N for the remaining 0.25 million cycles. The schematic view of a typical RCF setup is shown in Fig. 4. The shaft ends are mounted on SKF metal bearings, which are fixed in the pillow blocks. The bearing to be tested is mounted inside the bearing holder on the shaft, which is attached to one end of the lever. The loads are positioned at the other end of the lever. The accelerometer is placed on the joint of the bearing holder and lever. Furthermore, the bearing races were examined for morphology using FESEM after the test was conducted.

## Results and discussions

### Ball-on-disc test

**Fig. 5:**
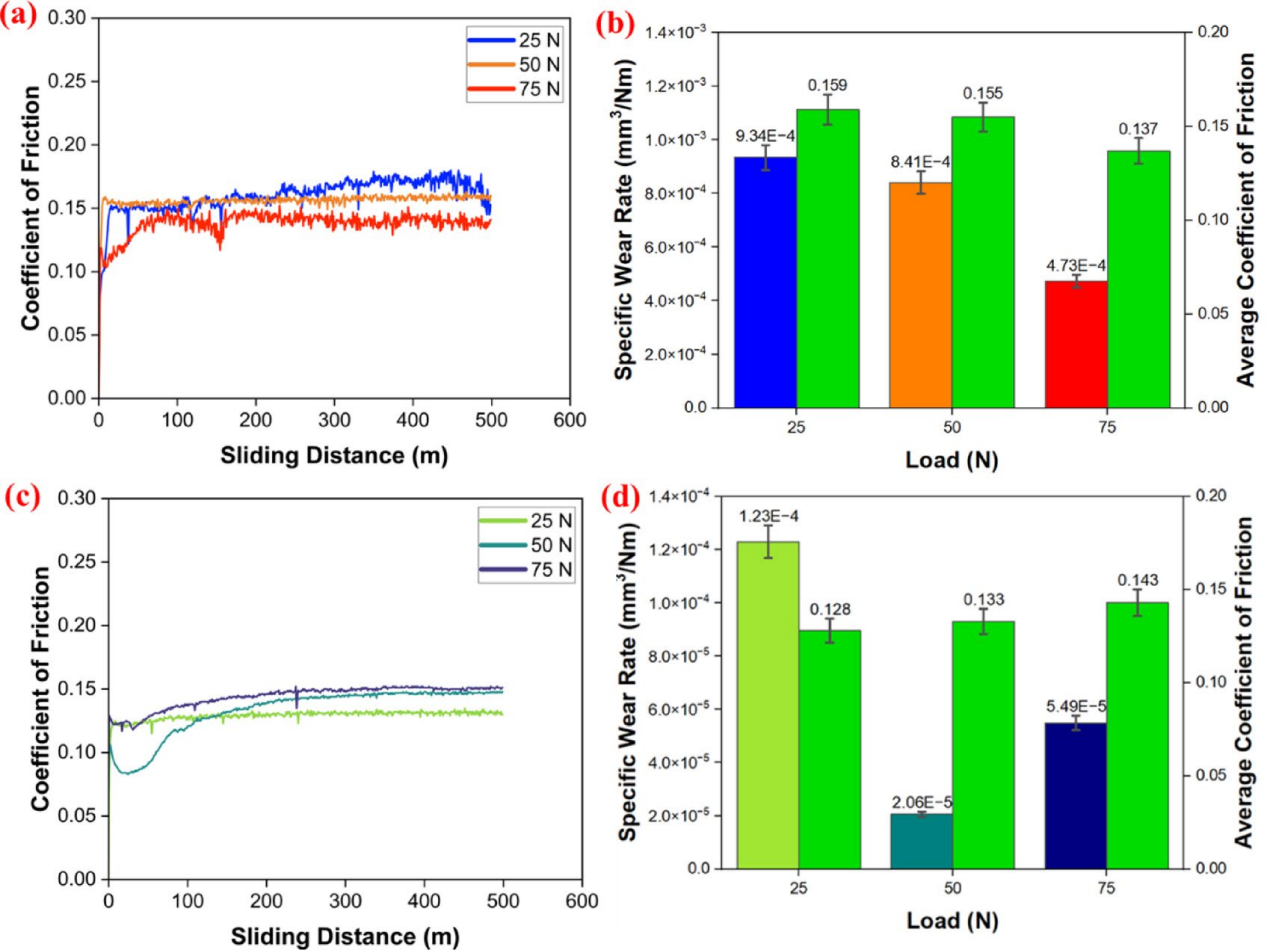
Friction and wear results; PTFE: (**a**) COF vs. sliding distance (**b**) specific wear rate and average COF. PEEK15 (**c**) COF vs. sliding distance (**d**) specific wear rate and average COF.

The disc speed was maintained at 800 RPM for a 20 mm wear track. For the loading systems of 25 N, 50 N and 75 N, friction and specific wear rate values were recorded, as shown in Fig. 5a–d. In the case of PTFE material, the average friction coefficient decreases as the load increases, and a similar trend is observed for specific wear rates^59^. Since, in the case of PTFE, an increase in load leads to effective transfer of film on the counter body, which results in polymer and polymer interactions, leading to a lower COF. Due to this, the lower COF obtained reduces the wear rate. This transfer of film is due to the adhesive wear of PTFE on the counter body. However, in the case of PEEK15, the COF values rise with the load. This is because PEEK, being a hard material, may cause the transfer film to be removed due to an increase in the contact area. Further, this ineffective transfer film increases the specific wear rate with an increase in load.

**Fig. 6:**
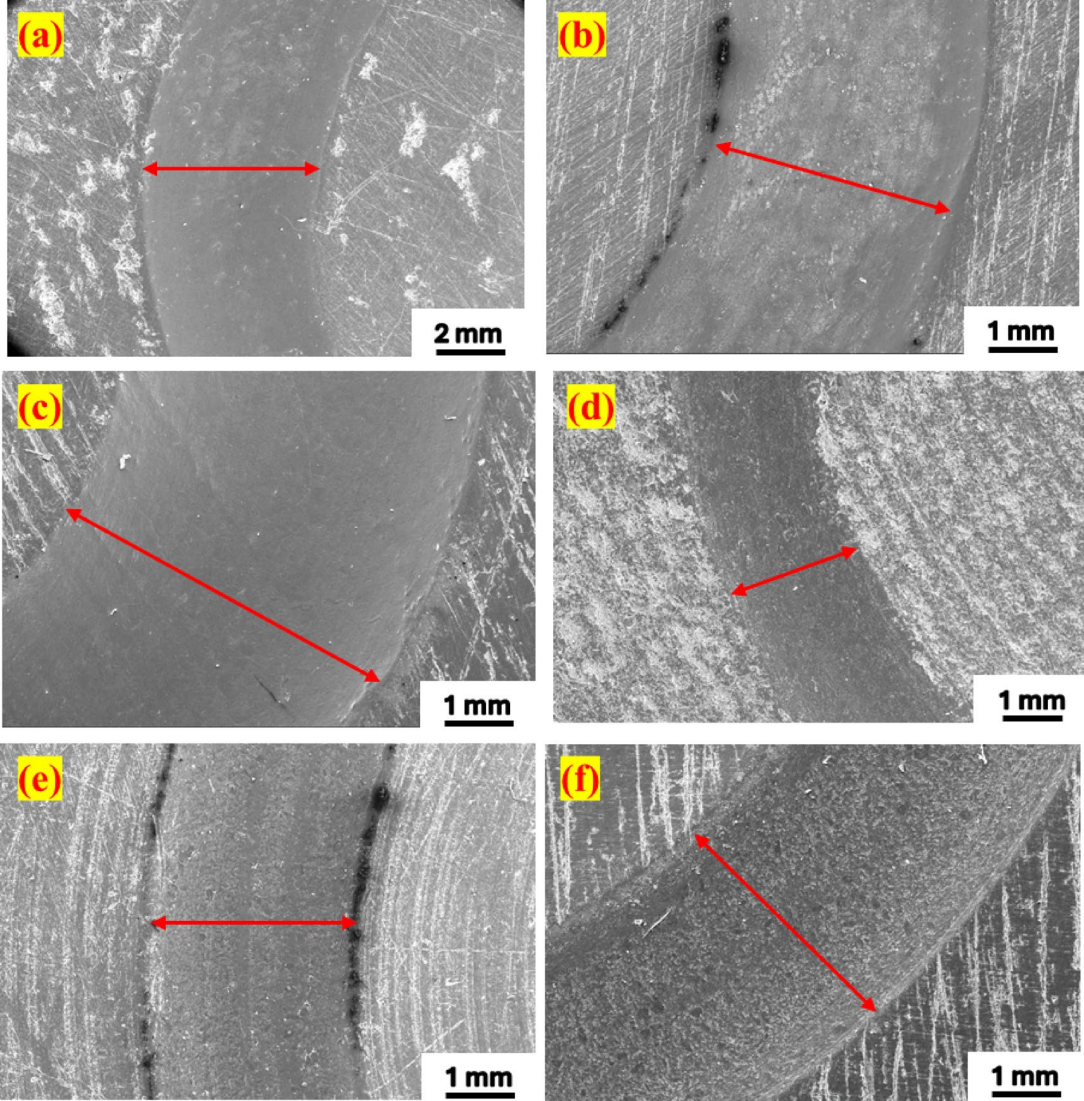
Surface morphology of wear track through FESEM; PTFE: (**a**) 25 N (**b**) 50 N (**c**) 75 N. PEEK15: (**d**) 25 N (**e**) 50 N (**f**) 75 N.

The result shows that PTFE becomes 86% more wear-resistant due to the reinforcement of PEEK. Overall, the results show that PEEK15 outperformed PTFE. The wear track surface was observed and analysed with FESEM. The wear track topography shows that as the load increases, the wider wear track is observed for PTFE and PEEK15, as shown in Fig. 6a–f. For both materials, increasing the load results in a larger contact area between the ball and disc, leading to a wider wear track during testing. At 25 N, the wear track forms primarily due to material delamination. In contrast, at 50 and 75 N, wear tracks result mainly from disc deformation, which is due to the high hardness of the silicon nitride ball. SEM measurements reveal that the wear track width on the PEEK15 disc is nearly 50% smaller than that on PTFE. These BOD test results indicate that pure PTFE may be unsuitable for ball bearing applications at higher loads.

### Free run test

**Fig. 7:**
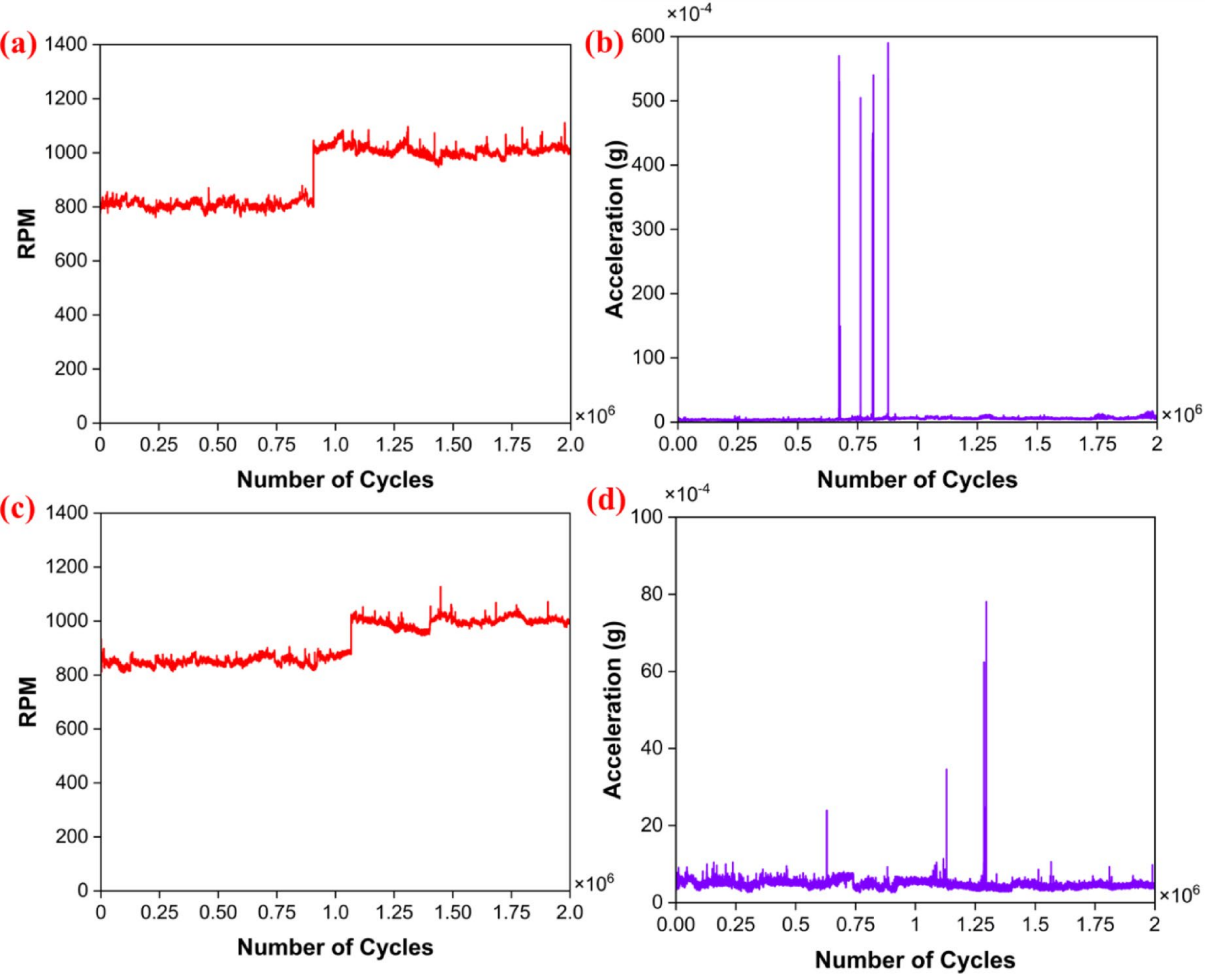
RPM and vibrational amplitude signals vs the number of cycles for PTFE (**a**, **b**) and PEEK15 (**c**, **d**).

Initially, the PTFE bearing was tested at 800 RPM for one million cycles. The results indicate stable RPM and vibrational amplitude values. Based on these consistent findings, it is evident that the rotor is intended to operate optimally at a speed of 1000 RPM. Furthermore, the results demonstrate improved stability in both the vibrational signals and RPM readings as compared to the previous one million cycles^59^. Overall, the results of the PTFE bearings indicate stable vibration amplitude (Figs. 7a–b). Although some peaks were observed at a certain speed due to wear. Throughout the test duration, the amplitude vibration was on the lower side. This may be due to the lower hardness of the PTFE materials and the balls used. Furthermore, it was observed that the speed remained stable up to 1 million cycles. However, some fluctuations were observed when the RPM was raised to 1000 RPM after one million cycles, as shown in Fig. 7b. For PEEK15, the bearing was rotated at 850 RPM up to 1 million cycles (Fig. 7c–d), and then the results obtained were favourable. Similarly, it rotated at 1000 RPM for an additional 1 million cycles, based on stable results, and promising outcomes were obtained. The speed was well in control, and the amplitude of vibrations was less than that observed in PTFE bearings.

These enhanced results are primarily due to the reinforcement of PEEK in the PTFE matrix that improves the mechanical properties, especially the hardness of the PTFE. Table 2 represents the mass loss in bearings. Among all the bearings, the PTFE bearing has the highest mass loss. Since PTFE and PEEK15 bearings were fabricated through the machining process, and during operation, the machine marks were removed, resulting in mass loss. Overall, the free-run test results indicate that PEEK15 exhibits superior performance compared to other bearings.

**Table 2:** Mass loss in bearings.

Bearings	Location	Before test (gm)	After 1 million			After 2 million		
			RPM	Wt. (gm)	Loss (gm)	RPM	Wt. (gm)	Loss (gm)
PTFE	Pillow Block 2	5.7940	800	5.6681	0.1259	1000	5.6164	0.1776
	Pillow Block 1	5.7820		5.7447	0.0373		5.6564	0.1256
PEEK15	Pillow Block 2	5.5510	850	5.4392	0.1118	1000	5.4365	0.1145
	Pillow Block 1	5.5448		5.5445	0.0003		5.5426	0.0022

**Fig. 8:**
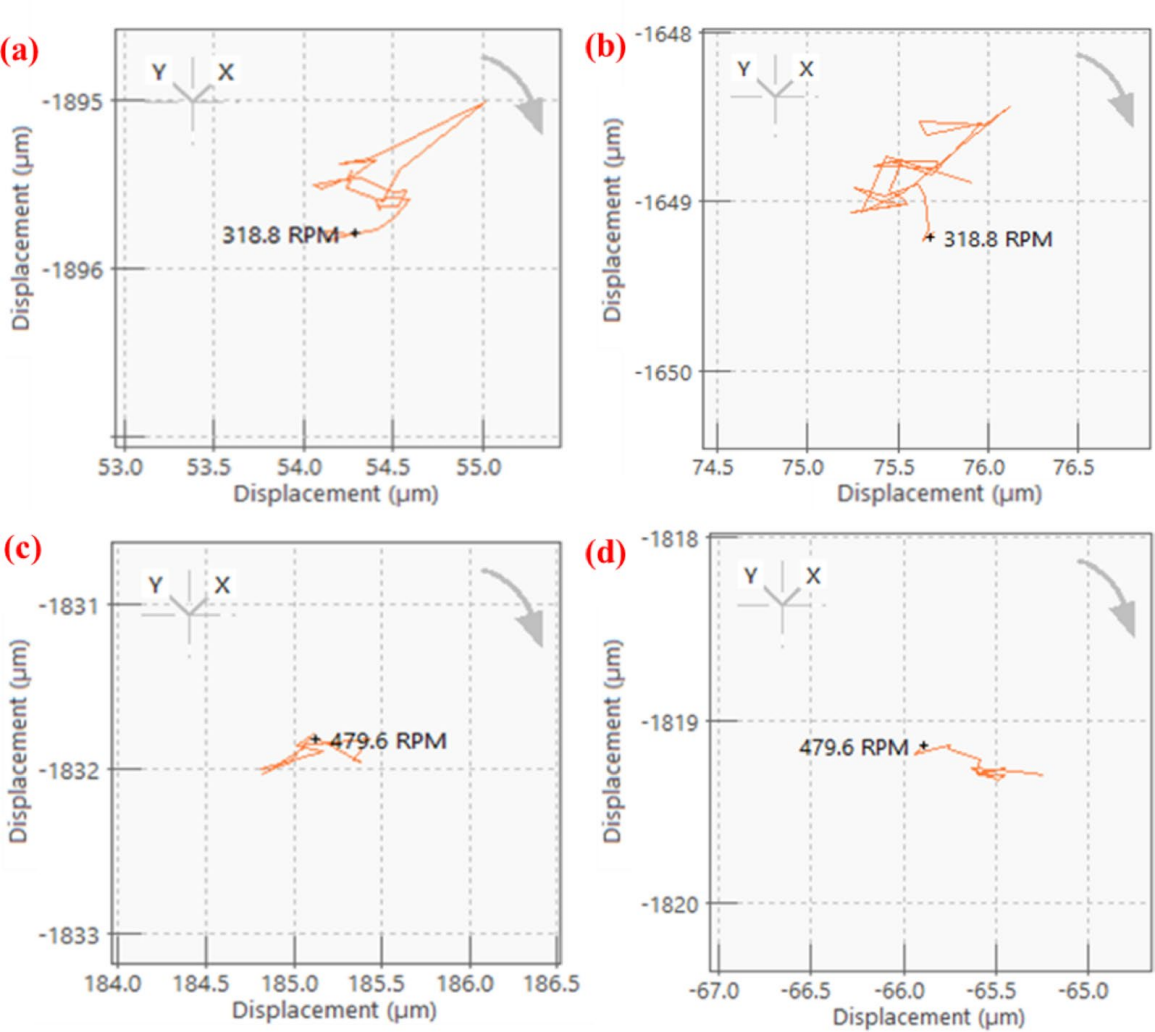
Shaft centre line plots at 650 RPM; PTFE: (**a**) pillow block 2 (**b**) pillow block 1; PEEK15: (**c**) pillow block 2 (**d**) pillow block 1.

**Fig. 9:**
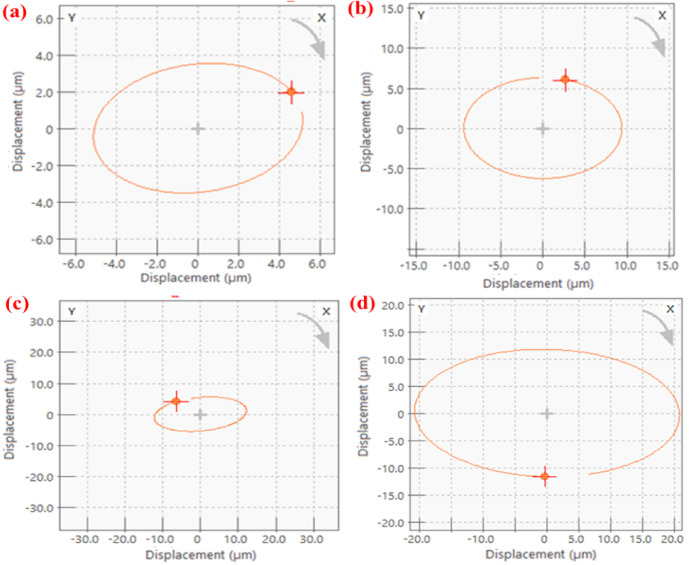
Orbit plots at 650 RPM; PTFE: (**a**) pillow block 2 (**b**) pillow block 1; PEEK15: (**c**) pillow block 2 (**d**) pillow block 1.

In the free run test, vibration analysis is performed after completing one million cycles. The shaft, supported on the bearings, was rotated from rest to its maximum speed. PTFE and PEEK15 were rotated up to a maximum speed of 1000 RPM. In this analysis, shaft centre line and orbit plots were obtained in the ORBI gate of OR35-INST, a 6-channel DAQ system. The orbit and shaft centreline are important plots for vibration analysis of the systems. These plots indicate the presence of unbalance, fault, or misalignment in the system. These plots were represented at a speed of 650 RPM. The shaft centreline plots (Fig. 8) show the displacement of the shaft centre in the XY plane. The displacement of the shaft or rotor centre during operation was approximately 0.5 µm, indicating no misalignment in the system. Further, the orbit plots represent the position of the shaft centre in the X and Y directions during one rotation of the shaft. Figure 9 shows the plots obtained for the rotor mounted with different sets of bearings. These are generally in an elliptical shape and depend on the system’s configuration. The plot for all the bearing sets is elliptical in shape. This may be because of less stiffness of the bearings. Overall, the dynamic characteristics indicate that there was no unusual behaviour in the rotor system and no defect or failure occurred in the bearing system.

## Rolling contact fatigue test

**Fig. 10:**
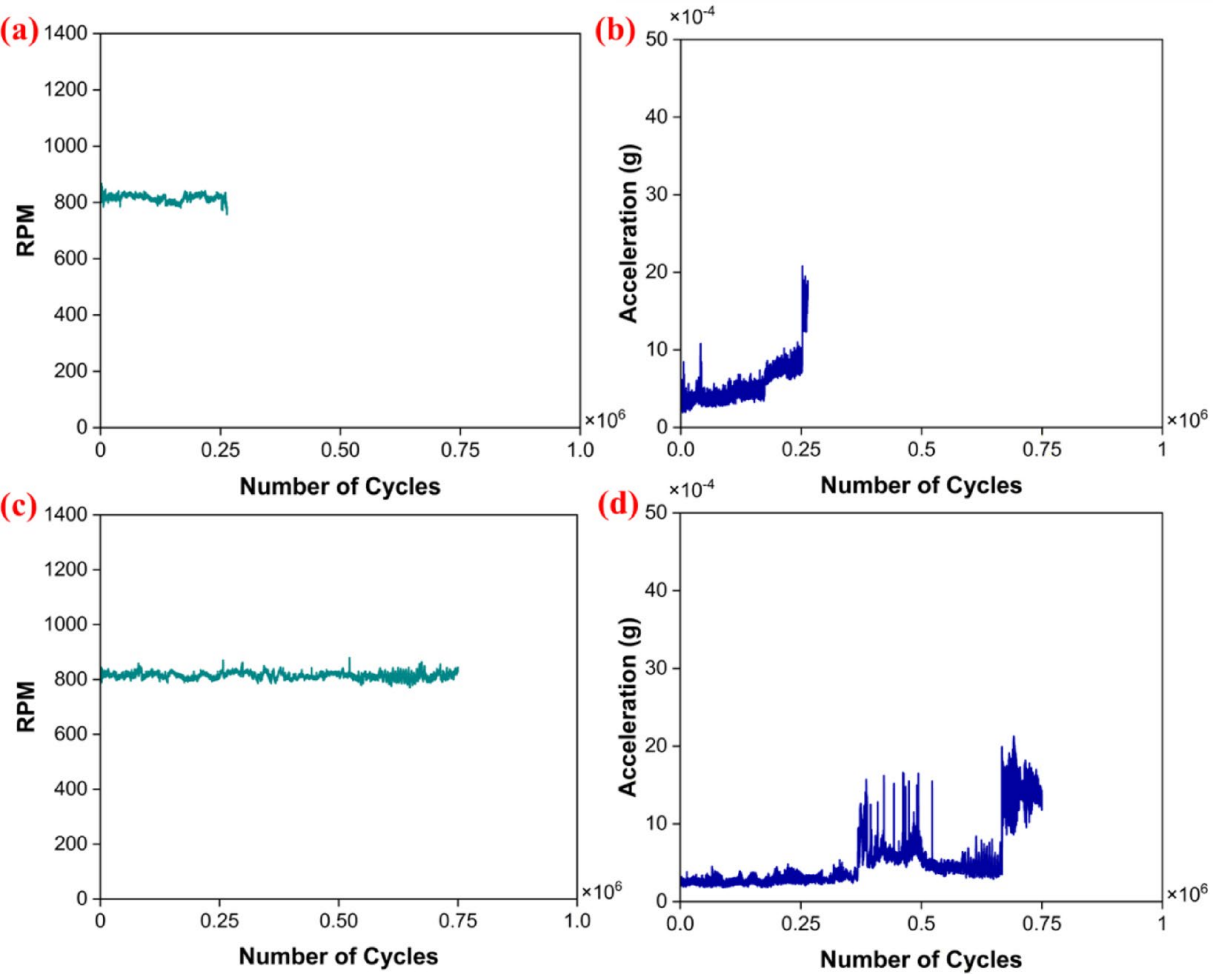
RCF results vs number of cycles; PTFE (**a**) RPM (**b**) vibrational amplitude. PEEK15 (**c**) RPM (**d**) vibrational amplitude.

New bearings of PTFE and PEEK15 were taken for the test. During the test, RPM and vibration signals were recorded. Initially, the PTFE bearing was tested, and the results reveal that the PTFE bearing ran smoothly up to 0.25 million rotations under a load of 36 N and failed at 2,630,000 cycles when subjected to a 72 N loading (Fig. 10a–b). The sudden rise in the vibrational amplitude (Fig. 10b) indicates the usual behaviour in bearing^59^. Later, the PEEK15 bearing was tested. The bearings ran smoothly under the loads of 36 N and 72 N up to 0.75 million cycles (Fig. 10c–d).

**Fig. 11:**
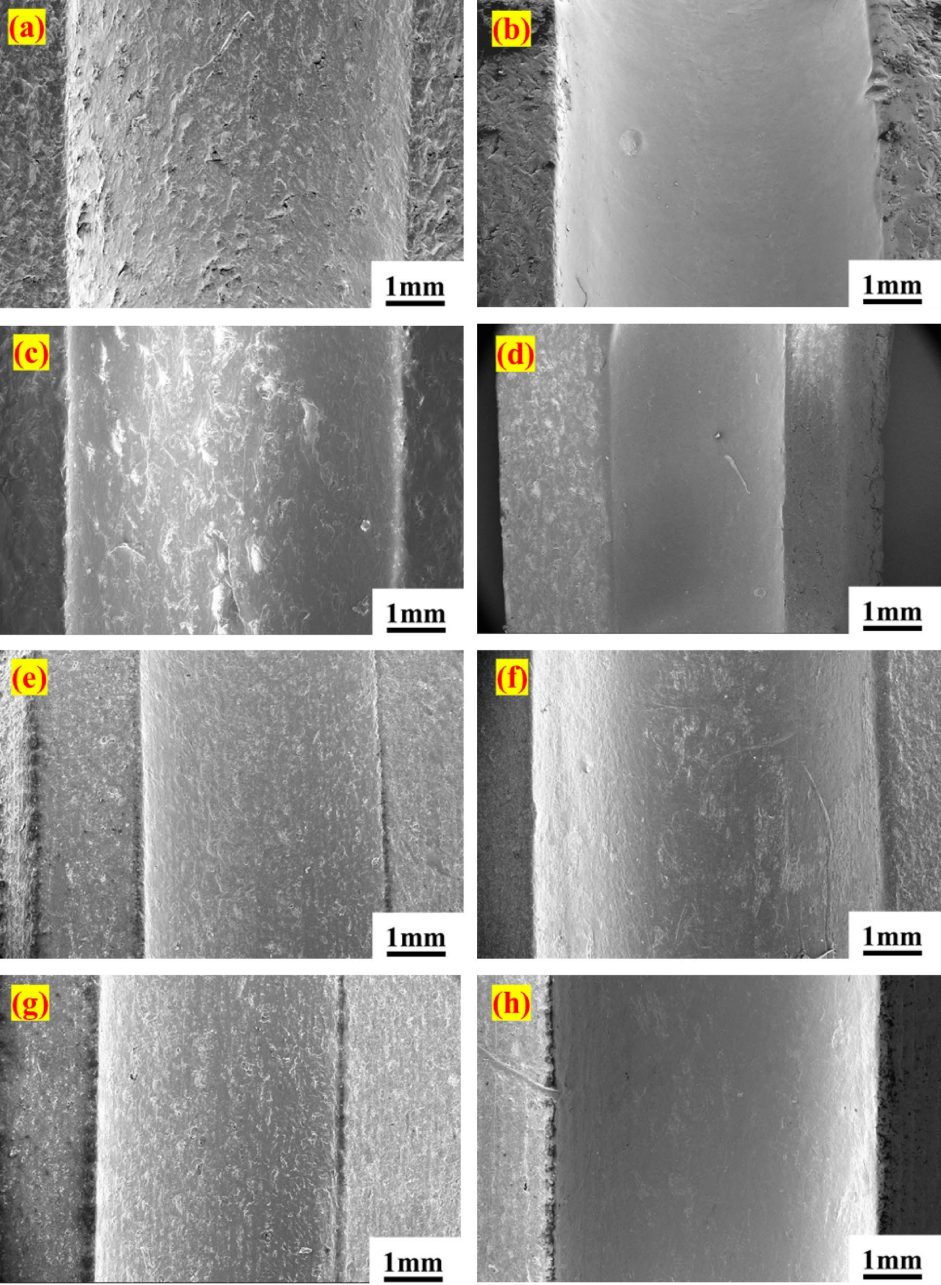
FESEM images of inner and outer races of the bearings; PTFE: (**a**) inner race before the test (**b**) inner race after failure (**c**) outer race before the test (**d**) outer race after failure; PEEK15: (**e**) inner race before the test (**f**) inner race after failure (**g**) outer race before the test (**h**) outer race after failure.

Before subjecting the bearing to a higher load, the condition was monitored, and it was observed that a certain gap had developed between the bearing races and the ball. Hence, bearing was declared failed. However, it has been observed that there was no cartographic failure in both bearings. However, the FESEM image reveals that the primary cause of the failure is the enlargement of the inner and outer ring races of the bearing (Fig. 11a–h). The widening of the bearing race is mainly observed in the bearing inner ring race. In the PTFE bearing, the inner ring race widened by almost 17%, and in the case of PEEK15, it was 21%. This widening of the bearing race is primarily due to the bearing ball’s high hardness and the PTFE composites’ low hardness. Due to this, the bearings became loose, exhibited undesirable vibration signals, and could not run at the desired speed. Although mass loss in the bearing has been noted, it is less than 1% in both bearings (Table 3).

**Table 3:** Mass loss during RCF test.

Bearing	Weight (gm)		
	Before	After	Loss
PTFE	5.5290	5.4760	0.053
PEEK15	5.8070	5.7408	0.0662

The RCF results of PTFE and PEEK15 indicate a sudden rise in vibration amplitude after a specific number of cycles. The testing was halted when this increase became persistent, signalling that

the bearing had failed. Additionally, fluctuations in RPM were observed; as the groove widened, the bearing’s stability diminished, preventing it from maintaining its speed. Notably, both bearings could withstand a load of 36 N, while PEEK15 demonstrated the ability to endure a load of 72 N. This suggests that reinforcement has significantly enhanced the bearing’s lifespan to a certain degree.

## Conclusions

In this article, two PTFE-based ball bearings with Si_3_N_4_ balls were fabricated to check their usability for bearing applications. The following are the conclusions of the proposed research work.

- The tribological test shows that a low friction coefficient (0.14–0.16) is maintained, which is feasible for the bearing application.
- The free run test shows that there is no misalignment or defect in the rotor systems after completion of 2 million cycles. Furthermore, the findings reveal that PEEK15 bearings exhibit greater stability than PTFE.
- Vibration characteristic results show that PTFE and PEEK bearings have shown excellent functionality under minimal load conditions, and there were no misalignments and faults in the rotor systems.
- RCF test results show that PTFE bearing fails at 0.26 million cycles, while PEEK15 fails at 0.75 million cycles.
- In both free run and RCF tests, it’s observed that the mass losses of the bearings were minimal.
- Bearing fabricated through the moulding process would be preferable. Since moulding processes are cost-effective, they could be used only in mass production.
- Metal reinforcement can be considered in the future since the reinforcement of metal can significantly reduce the wear rate.
- There is scope to develop polymer bear
- ings through additive manufacturing, especially the bearing cages.
- The cost of the bearing is still higher than or almost comparable to that of a metal bearing. At the same cost, polymer-based ball bearings may not offer the same performance as metal bearings.

It can be concluded that the PTFE bearing with Si_3_N_4_ balls is not suitable for ball-bearing applications. But PEEK15 is suitable for loads up to 50 N. The reinforcement of PEEK in PTFE has significantly improved its overall tribological performance. Suitable reinforcements must be selected to improve the bearing performance, including metals, ceramics, and carbon-based materials, which enhance their hardness and wear resistance.

## Data Availability

Data to support the findings of this study are available with the corresponding author and made available upon reasonable request.
